# Evolution from viral encephalitis to autoimmune encephalitis to multiple sclerosis: a case report

**DOI:** 10.1007/s00415-024-12659-9

**Published:** 2024-08-31

**Authors:** Katharina Wurdack, Harald Prüss, Carsten Finke

**Affiliations:** 1 Department of Neurology, Charité Universitätsmedizin Berlin, Berlin, Germany; 2 German Center for Neurodegenerative Disorders Berlin, Berlin, Germany; 3 Berlin School of Mind and Brain, Humboldt-Universität zu Berlin, Berlin, Germany

**Keywords:** Viral encephalitis, Autoimmune encephalitis, Multiple sclerosis, Neuroimmunology, Overlap syndrome, Case report

## Abstract

**Background:**

There are established associations between viral and autoimmune encephalitis as well as between autoimmune encephalitis and demyelinating central nervous system (CNS) diseases. Here, we report the evolution from varicella zoster virus (VZV) encephalitis to limbic autoimmune encephalitis (AIE) to multiple sclerosis (MS) in one patient.

**Case report:**

A woman in her mid-thirties presented with headache, aphasia, and a generalized tonic–clonic seizure. Cerebrospinal fluid (CSF) VZV polymerase chain reaction was positive and treatment with acyclovir was administered for VZV encephalitis. Five months later, the patient presented with cognitive deficits and MRI showed new bilateral hippocampal T2-hyperintensities. CSF analyses revealed pleocytosis and neuropil antibodies in tissue-staining. A diagnosis of limbic AIE was established and treatment with IV steroids and IV immunoglobulins initiated. One year later, the patient developed paresthesia of both legs and magnetic resonance imaging studies now showed new supratentorial and spinal demyelinating lesions. The patient was diagnosed with MS and treatment was changed to rituximab.

**Conclusions:**

This unique case report links three important neuroimmunological entities in characterizing the evolution from infectious to autoimmune encephalitis to multiple sclerosis in one patient. Identification of such rare clinical constellations is critical for correct treatment choice and provides important novel insights into the pathophysiology of neuroimmunological disorders including viral triggers and overlap manifestations of autoimmune CNS diseases.

## Introduction

Autoimmune encephalitis (AIE) can be triggered by viral infections of the CNS, which is best described for herpes simplex virus encephalitis (HSVE) [1]. A recent study showed that up to 27% of HSVE patients develop AIE, of which around half manifest as encephalitis with antibodies directed against the NMDA receptor (NMDARE) and half as encephalitis with antibodies against unknown neural epitopes [2]. Additional suspected viral triggers of AIE include the varicella zoster virus (VZV) [3–5]. Furthermore, overlap syndromes between NMDARE and demyelinating diseases such as multiple sclerosis (MS), neuromyelitis optica spectrum disorder (NMOSD), and myelin oligodendrocyte glycoprotein antibody-associated disease (MOGAD) are increasingly reported and manifest in up to 3% of NMDARE patients [6, 7]. Here, we report on a patient with VZV encephalitis and subsequent limbic AIE with neuropil antibodies, followed by MS.

## Case report

**Fig. 1:**
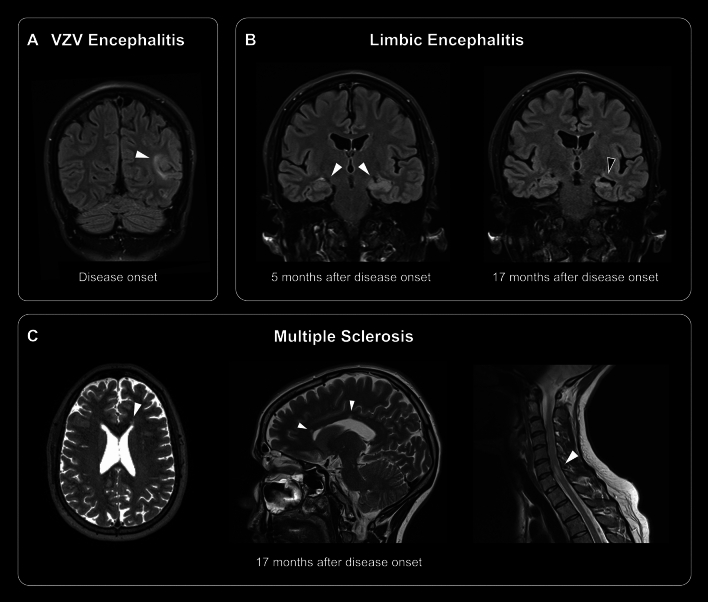
Cerebral and spinal MRI findings. At onset of VZV encephalitis, a hyperintense lesion under the left angular gyrus was present (panel **A**). Five months later, limbic encephalitis manifested with bilateral (left > right) hippocampal FLAIR hyperintensity, which reverted to left hippocampal atrophy under immunomodulating treatment (panel **B**). Seventeen months after onset of VZV encephalitis and twelve months after onset of limbic AIE, new periventricular, callosal, and spinal T2 hyperintense lesions supported the additional diagnosis of multiple sclerosis (panel **C**). White arrows indicate FLAIR hyperintense lesions, black arrow indicates atrophy. *AIE* autoimmune encephalitis; *MRI* magnetic resonance imaging; *FLAIR* fluid attenuated inversion recovery; *VZV* varicella zoster virus

A woman in her mid-thirties without any relevant previous medical history presented with sudden-onset motor aphasia followed by a generalized epileptic seizure, and recurring headaches accompanied by photo- and phonophobia as well as difficulties concentrating during the last two months. Neurologic examination revealed expressive aphasia with impaired language processing. Routine blood tests were normal; MRI showed a left angular gyrus FLAIR hyperintensity (Fig. 1, panel A) without evidence for diffusion restriction. CSF analyses revealed increased white blood cell count (WBC, 21/µl), while red blood cell count (RBC, 2/µl), protein (419 mg/l), and lactate (1.33 mmol/l) were normal. Semiquantitative PCR from CSF was negative for HSV1/2, but positive for VZV, and anti-VZV-IgG was elevated. Serologically, acute infection with EBV, HIV, HCV, borreliosis, and syphilis were ruled out. OCB were positive in both serum and CSF (type 4). Testing for anti-NMDAR antibodies from CSF as well as anti-CASPR2 antibodies and anti-Hu antibodies from serum was negative. An angiogram showed no signs of vasculitis or vasculopathy. IV acyclovir was started with 1,000 mg every 8 h for VZV encephalitis and continued for 14 days, resulting in reduced headache severity and improved concentration. In addition, the patient was started on levetiracetam 1,000 mg daily and no further seizures occurred.

Five months after initial symptom onset, the patient was admitted for a focal seizure with aphasia, epigastric auras, concentration deficits, and memory impairment. CSF studies showed pleocytosis (WBC 15/µl), normal RBC (0/µl), protein (285 mg/l), and lactate (1.41 mmol/l) as well as negative VZV and HSV1/2 PCR. OCB were positive in CSF but not in serum (type 2). Extensive conventional testing for anti-neuronal autoantibodies in CSF and serum was negative. However, immunohistological mouse brain staining with CSF in our laboratory showed neuropil-directed autoantibodies. MRI revealed new bilateral (left > right) hippocampal FLAIR hyperintensities (Fig. 1, panel B). The patient was diagnosed with definite limbic AIE [8] and received treatment with IV methylprednisolone 1,000 mg daily for 5 days and IV immunoglobulins (150 mg total). Immunotherapy led to an improvement in cognitive function, headache severity, and seizures (in addition to adapted anti-seizure medication with levetiracetam 3,000 mg daily and lacosamide 200 mg) but was escalated to plasmapheresis given persistent fatigue, concentration difficulties, and headache. At this point, the patient was transferred to our hospital for further treatment after an anaphylactic reaction during the fifth plasmapheresis cycle.

Eighteen months after initial symptom onset (twelve months after onset of limbic AIE), the patient reported fluctuating leg paresthesia. Follow-up MRI revealed new T2/FLAIR hyperintense lesions suggestive of MS, i.e., lesions in the corpus callosum, periventricular lesions, and a spinal cord lesion (Fig. 1, panel C). Anti-AQP4 and anti-MOG-antibodies were negative in serum. The patient was referred to our neuroimmunological outpatient clinic and was diagnosed with MS upon fulfilling the McDonald criteria [9]. Treatment was now switched to 1,000 mg rituximab to address both AIE and MS, which improved fatigue severity, memory deficits, headache, hyperacusis, and paresthesia after six months. The patient also received cognitive training and benefitted from cognitive behavioral therapy after hospital discharge.

At last follow-up, the patient reported an improvement in overall symptom severity. Persisting symptoms included fatigue and intermittent headache. She lives independently and plans occupational rehabilitation. Cerebral and spinal MRI 36 months after first presentation showed persistent left hippocampal atrophy and constant MS-associated T2/FLAIR lesion load.

The patient has provided written informed consent to the submission of the case report to the journal. Approval was obtained from the ethics committee of Charité – Universitätsmedizin Berlin.

## Discussion

This case presents a unique clinical constellation, i.e., viral encephalitis followed by autoimmune encephalitis and multiple sclerosis. Diagnosis of VZV encephalitis was established based on the presence of headaches with photo- and phonophobia as well as concentration difficulties, CSF pleocytosis, positive semiquantitative VZV-PCR, and elevated VZV-IgG in CSF. MRI revealed a FLAIR-hyperintense angular gyrus lesion. Indeed, while MRI is often unremarkable in VZV encephalitis, up to 15% of patients develop post-inflammatory lesions of deep-gray matter or supratentorial white-matter structures [10]. Notably, VZV encephalitis in our patient followed a mild disease course. However, it has been suggested that due to underreporting of mild encephalitic manifestations, the frequency of VZV encephalitis may be underestimated [10]. Furthermore, viral triggers including HSV and VZV infections have been established in the pathophysiology of AIE [11]. Following HSVE, anti-neuronal autoantibodies are present in up to 42% of patients (around half of which are directed against unknown neural epitopes), and 23% of patients develop AIE within a median of 30–39 days. However, the observed delay between HSVE and onset of AIE ranges up to 510 days [2, 12], indicating significant variability in the time course of post-viral AIE manifestation. Similarly, several cases of NMDARE following other viral CNS infections including VZV encephalitis have been reported [3–5]. However, the exact mechanisms inducing the production of autoantibodies associated with AIE remain unclear. Potential factors include antigen-independent viral stimulation of B cells (e.g., via Toll-like receptors), molecular mimicry, or exposure to tissue destruction-related neoantigens [11]. Here, we report a novel association between VZV encephalitis and AIE with antibodies directed against unknown neuropil epitopes.

Overlap syndromes of AIE with demyelinating CNS diseases have almost exclusively been investigated in patients with NMDARE, where they have been reported in up to 3% of cases, exceeding the expected frequency based on reported prevalence rates for the most common demyelinating disorder, i.e., MS [13]. AIE can precede the first demyelinating episode or vice versa with a delay of up to several years between the two disorders, or both can occur simultaneously [6]. Importantly, the presence of isolated OCB in CSF can occur both in AIE as well as in MS, and is included in both the diagnostic criteria for autoantibody-negative probable AIE [8] and the diagnostic criteria for MS [9]. Future studies are needed to explore the dynamics of CSF parameters including OCB in larger patient cohorts with overlap syndrome. Most cases of overlap syndrome have been observed so far as association of NMDARE with MOGAD or NMOSD [6], although overlaps between NMDARE and MS were reported in a systematic literature review [7]. It seems plausible that an individual predisposition for autoimmune disorders is of relevance in the development of these overlap syndromes. In addition, viral infections might be shared triggers between demyelinating CNS diseases and AIE. For example, Eppstein-Barr virus infection has been suggested to play a causal role in MS pathophysiology through molecular mimicry with glial or myelin proteins and B cell induction [14, 15]. So far, only one case of limbic AIE without detection of known autoantibodies and concomitant MS has been reported, although no tissue-based testing for autoantibodies was conducted in this patient [16]. The current case thus confirms that overlap syndromes extend beyond NMDARE and should be considered in patients with AIE or demyelinating diseases presenting with atypical symptoms.

In conclusion, AIE should be considered in patients presenting with recurrent or new neurologic symptoms following infectious encephalitis. Post-infectious AIE will most often occur after HSVE, but can also manifest after other viral CNS infections and can arise in association with different autoantibodies. Patients exhibiting autoantibodies directed against unknown neural antigens have likely been underreported due to limited availability of tissue-based antibody-testing. The presence of these yet-uncharacterized antibodies in around one fifth of HSVE patients [2] underlines the importance of tissue-based testing in patients with suspected AIE and negative conventional antibody-testing. Importantly, established clinical criteria [8] should be employed to inform treatment decisions in suspected AIE. In addition, while overlap syndromes with demyelinating CNS diseases occur most often in association with NMDARE, they can likewise affect patients with other AIE variants. Importantly, AIE and demyelinating episodes can occur sequentially or simultaneously [6, 7], so increased awareness for atypical symptoms in patients with AIE or demyelinating CNS disease is warranted.

Concerning the pathophysiology of AIE and demyelinating CNS diseases, both external and individual factors must be considered. While many environmental risk factors for MS have been identified [17], data to address this question in AIE is still lacking due to the much lower incidence. However, both the development of AIE and demyelinating CNS disease have been associated with innate and acquired alterations of the immune system [11, 17]. Viral infections may represent an influential external element that, taken together with individual susceptibility to immune system dysregulation, could trigger neuroimmunological disease [2, 11, 14, 15]. The identification of exceptional cases such as our patient, with the transition from infectious to autoimmune neurologic disease and the additional development of demyelinating CNS disease, may help to increase our understanding of shared mechanisms of immune dysregulation across infectious and neuroimmunological diseases.

## Data Availability

Not applicable.

## References

[1] Prüss H et al (2012) N-methyl-D-aspartate receptor antibodies in herpes simplex encephalitis. Ann Neurol 72(6):902–911 23280840 10.1002/ana.23689 PMC3725636

[2] Armangue T et al (2023) Neurologic complications in herpes simplex encephalitis: clinical, immunological and genetic studies. Brain 146(10):4306–19 37453099 10.1093/brain/awad238

[3] Schäbitz WR et al (2014) VZV brainstem encephalitis triggers NMDA receptor immunoreaction. Neurology 83(24):2309–2311 25378669 10.1212/WNL.0000000000001072

[4] Prakash PA et al (2019) Anti-NMDAR encephalitis with concomitant varicella zoster virus detection and nonteratomatous malignancy. Neurol Neuroimmunol Neuroinflamm 6(2):e537 30697587 10.1212/NXI.0000000000000537 PMC6340333

[5] Fatma N et al (2022) Atypical anti-NMDA receptor encephalitis associated with varicella zoster virus infection. J Neurovirol 28(3):456–459 35604574 10.1007/s13365-022-01080-5

[6] Titulaer MJ et al (2014) Overlapping demyelinating syndromes and anti–N-methyl-D-aspartate receptor encephalitis. Ann Neurol 75(3):411–428 24700511 10.1002/ana.24117 PMC4016175

[7] Zhang S et al (2022) Clinical characteristics of anti-N-methyl-d-aspartate receptor encephalitis overlapping with demyelinating diseases: a review. Front Immunol 13:857443 35837405 10.3389/fimmu.2022.857443 PMC9273846

[8] Graus F et al (2016) A clinical approach to diagnosis of autoimmune encephalitis. Lancet Neurol 15(4):391–404 26906964 10.1016/S1474-4422(15)00401-9 PMC5066574

[9] Thompson AJ et al (2018) Diagnosis of multiple sclerosis: 2017 revisions of the McDonald criteria. Lancet Neurol 17(2):162–173 29275977 10.1016/S1474-4422(17)30470-2

[10] Kennedy PGE (2023) The spectrum of neurological manifestations of varicella-zoster virus reactivation. Viruses 15(8):1663 37632006 10.3390/v15081663 PMC10457770

[11] Prüss H (2021) Autoantibodies in neurological disease. Nat Rev Immunol 21(12):798–813 33976421 10.1038/s41577-021-00543-w PMC8111372

[12] Cleaver J et al (2024) The immunobiology of herpes simplex virus encephalitis and post-viral autoimmunity. Brain 147(4):1130–1148 38092513 10.1093/brain/awad419 PMC10994539

[13] Walton C et al (2020) Rising prevalence of multiple sclerosis worldwide: Insights from the Atlas of MS, third edition. Mult Scler 26(14):1816–1821 33174475 10.1177/1352458520970841 PMC7720355

[14] Bjornevik K et al (2022) Longitudinal analysis reveals high prevalence of Epstein-Barr virus associated with multiple sclerosis. Science 375(6578):296–301 35025605 10.1126/science.abj8222

[15] Lanz TV et al (2022) Clonally expanded B cells in multiple sclerosis bind EBV EBNA1 and GlialCAM. Nature 603(7900):321–327 35073561 10.1038/s41586-022-04432-7 PMC9382663

[16] Karaaslan Z et al (2017) A case of seronegative limbic encephalitis with multiple sclerosis: a possible overlapping syndrome. Am J Case Rep 18:64–66 28096524 10.12659/AJCR.901391 PMC5266202

[17] Reich DS, Lucchinetti CF, Calabresi PA (2018) Multiple sclerosis. N Engl J Med 378(2):169–180 29320652 10.1056/NEJMra1401483 PMC6942519

